# Parity and hypertension risk in couples: does number of parity matter: findings from Tehran Lipid and Glucose Study

**DOI:** 10.1186/s12889-023-15397-1

**Published:** 2023-03-13

**Authors:** Maryam Rahmati, Marzieh Saei Ghare Naz, Fereidoun Azizi, Fahimeh Ramezani Tehrani

**Affiliations:** 1 Reproductive Endocrinology Research Center, Research Institute for Endocrine Sciences, Shahid Beheshti University of Medical Sciences, Tehran, Iran; 2 Endocrine Research Center, Research Institute for Endocrine Sciences, Shahid Beheshti University of Medical Sciences, Tehran, Iran

**Keywords:** Parity, Children, Blood pressure, Hypertension, Metabolic syndrome, Couple

## Abstract

**Background and aims:**

As reported, hypertension (HTN) plays a leading role in explaining mortality worldwide, but it still has many confounding factors. This study explored whether the number of parity and age matters for HTN among couples from the Tehran Lipid and Glucose Study (TLGS).

**Methods:**

This study was conducted on 2851 couples from TLGS. All the variables were collected based on the standard protocol. The participants were categorized into four and five categories according to the number of parity (childless, one, two, three, or more parities) and age (18-30y, 30-40y, 40-50y, 50-60y, and 60-70y), respectively. Spline regression models via log link function for the binary outcome and linear link function for continuous outcomes were applied to evaluate the effect of interaction term age and parity categories on the desired outcome.

**Results:**

Among the total of 2851 pairs, 2.3% had no child, 9.5% had 1 child, 38.4% had 2 children, and 49.8% had ≥ 3 children. The adjusted risk (95% CI) of HTN in females aged 40-50y with 1 child, 2 and ≥ 3 children compared to no child were 1.14(1.04, 1.26), 1.05(1.01, 1.10), 1.12(1.07, 1.17), respectively (*p* < 0.05). Moreover, in those aged 50-60y with 2 and ≥ 3 children, the risk of HTN significantly increased by 4%. In females aged 60-70y with ≥ 3 children compared to those without children, the risk of HTN increased by 2%. For males aged 30-40y with 2 children compared to the no child group, the adjusted risk of HTN increased by 17%, while for those with ≥ 3 children in the same age group, this risk significantly decreased by 13%. Moreover, in males aged 30-40y with 2 children, risk ratio of HTN increased by 17%, but in males with ≥ 3 children, it decreased by 13% and in those in the same groups but aged 40-50y the risk increased by 6% and 11%, respectively.

**Conclusion:**

Our findings suggest that gender, childlessness, having one child, and multi-parity had different impacts on HTN. Further research is needed to confirm our findings.

## Highlights

• Gender, childlessness, having one child, and multi-parity had different impacts on HTN.

• The increased risk of HTN was observed among females aged 40-50y with 1 child, 2 and ≥ 3 children compared to females with no child.

• In females aged 50-60y with 2 and ≥ 3 children, and in females aged 60-70y with ≥ 3 children the risk of HTN increased compared those without children.

• In males aged 30-40y with 2 children compared to the no-child group the risk of HTN increased.

## Introduction

Hypertension (HTN) is one of the most prevalent risk factors for non-communicable diseases (NCD) [1]. It represents no warning signs or symptoms, so is mainly known as a silent killer [2]. HTN per se acts as the main risk factor for atherosclerosis, renal disease, stroke, and peripheral arterial diseases [2]. Although the exact cause of HTN in most cases is unknown [3], it is well established that the conditions that increase a person's risk of developing HTN include genetic factors, ageing, stress, unhealthy diet, physical inactivity, tobacco, and alcohol use, obesity, and pollution [3, 4]. Further, reproductive factors have been found to be associated with HTN [5].

Parity has long been seen as a condition primarily affecting females. However, childbearing could also affect the health status of males. Metabolic and hormonal factors alter during normal pregnancy [6]. Apart from the link between physiological and pathological changes during pregnancy and the later development of diseases [7, 8], other factors such as behavioral patterns related to childbearing could affect the health of parents [9, 10]. Recent studies have shown that childbearing may affect males’ and females’ health. The previous meta-analysis demonstrated the J-shaped dose–response relationship between a number of parity and cardiovascular diseases (CVD) [11]. Previous studies have indicated that the number of parity may also be associated with cognitive function [12], HTN [13], and chronic kidney disease (CKD) [14]. Some part of this association was explained by biological alterations in the endocrine and immune system during pregnancy [14]. As family members share a common environment and are under similar circumstances, genetic factors are an important factor that could influence health [15]. Consequently, having children may lead to significant health changes over the life course in both females and males.

Thus far, limited prior studies have investigated the association between the number of parity and HTN risk in females [16–18]. These studies have yielded conflicting results. Despite the growing number of studies, controversy still exists about positive or inverse associations between the number of parity and HTN among females and males. Gender is a matter in hypertension development and it is well established that males had consistently higher blood pressure (BP) and were at greater risk for HTN [19, 20]. Hormonal differences play an important role in these gender differences [20]. In this study, we strive to illustrate the impact of number of parity on the risk of HTN in females and males in different age groups. To separate the non-biological effects of parenthood from the biological effects of pregnancy, we also investigated the effects of fatherhood on HTN risk. Recognizing the high-risk group of couples during different life stages could provide an opportunity for family-based interventions.

## Method

This study was conducted using data from Tehran Lipid and Glucose Study. The protocol of this prospective cohort study was designed according to the World Health Organization (WHO)-recommended model for non-communicable diseases (NCD) surveillance [21]. The population of the TLGS study was selected by multistage stratified cluster random sampling technique from urban district 13 of Tehran, the capital of the Islamic Republic of Iran. This population was representative of the overall population of Tehran at the beginning (1999–2001) of the study. Informed written consent was obtained from all the participants. In this study, all the family members were invited to participate in the study. In total, 15,005 participants aged ≥ 3y were invited to the TLGS-specific data-gathering center. A detailed description of the rationale, design, and methodology of the TLGS study has been previously published [22].

All methods were carried out in accordance with relevant guidelines and regulations with the Declaration of Helsinki Informed consent was obtained from all subjects.

### Study population

In this analysis, the participants were selected from the last examination phase (2015–2018) of the TLGS. For the current study, 2851 couples were included in the analysis. The age variable ranged from 18 to 70 years and was categorized into five groups (18-30y, 30-40y, 40-50y, 50-60y, and 60-70y).

### Measures

All the participants were studied by trained physicians according to the standard protocol. Information for demographic and clinical variables was obtained using a standard and validated questionnaire. Smoking status in this study was considered as past, current, and never user. Anthropometric, laboratory, and clinical assessments were performed based on the TLGS measurement protocol [22]. All the blood analyses were carried out at the TLGS research laboratory. Details of the laboratory measurements, including fasting blood glucose (FBS) levels, triglyceride (TG), low-density lipoprotein cholesterol (LDL-C), high-density lipoprotein cholesterol (HDL-C), and total cholesterol (TC), have been reported previously [22].

Blood pressure (BP) was measured after a 15-min rest in the sitting position; moreover, two systolic and diastolic blood pressure measurements were taken on the right arm using a standardized mercury sphygmomanometer (calibrated by the Iranian Institute of Standards and Industrial Researches). Hypertension was defined as hypertension diagnosed by a physician, the current use of antihypertensive drugs, or systolic blood pressure (SBP) of ≥ 140 mmHg or final diastolic blood pressure (DBP) of ≥ 90 mmHg [23].

Parity is defined as the number of live births for females and the number of children for males. This information was gathered through individual interviews at the time of the survey. Based on the evidence, the self-reported number of parity had high validity [24]. For the data analysis of the present study, the number of parity was categorized into four groups (0, 1, 2, and ≥ 3).

### Statistical analysis

Continuous variables were checked for normality based on the one-sample Kolmogorov–Smirnov test and were presented as mean (standard deviation) if they had a normal distribution, or median with an inter-quartile range (IQ25-75) for the variables with skewed distribution. Categorical variables were presented in number and percentage. Characteristics of the participants were compared between the parity categories by applying ANOVA or $\chi^2$ test for continuous and categorical data, respectively. Appropriate postdoc analysis was used for pairwise comparisons. The Kruskal–Wallis test was applied to compare the variables with skewed distribution. The spline regression models via log link function for the binary outcome and linear link function for continuous outcomes were applied to evaluate the effect of interaction term age and parity categories on the desired outcome. The marginal means for SBP and DBP and marginal probability for HTN status were plotted for both males and females based on the different categories of age. These models were also adjusted for the potential risk factors: BMI, smoking status, physical activity, education, TG, and HDL. We used covariate-adjusted spline regression within a cross-sectional framework. This model allows for flexible consideration of non-linear age-associated patterns while accounting for traditional covariates and interaction effects. Statistical analysis was performed using the software package STATA (version 13; STATA Inc., College Station, TX, USA) and the significance level was set at *P* < 0.05.

## Result

**Fig. 1:**
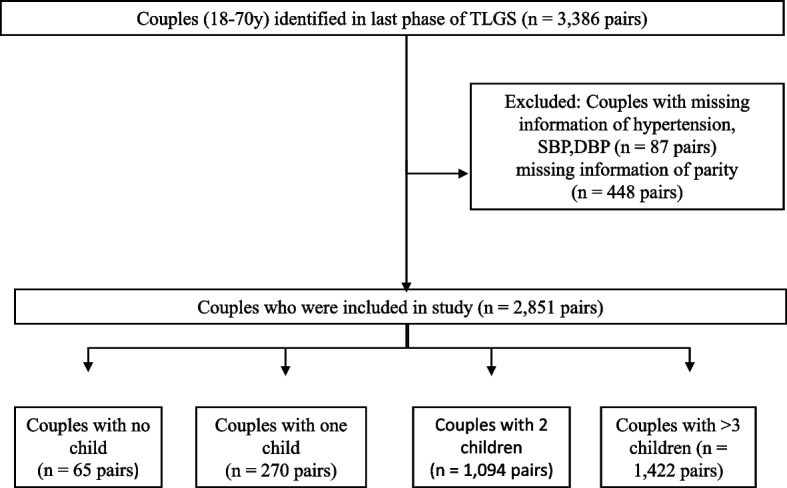
Flowchart of the study. *Abbreviations: TLGS, Tehran lipid and glucose study; SBP, systolic blood pressure; DBP: Diastolic blood pressure

Of a total of 2851 pairs, aged 18–70 years, 65 (2.3%) had no child, 270 (9.5%) had 1 child, 1094 (38.4%) had two children and 1422 (49.8%) had 3 or more children (Fig. 1). In this study, there were significant differences between the mean (SD) ages of females with no child 38.5(14.8), and females with two children 45.4 (8.1) and three or more children 53.0(8.5) (*p* < 0.05). Similarly, there were significant differences between the mean (SD) ages of males with no child 44.4(15.4) and males with two 50.6(8.4) and three or more children 58.1(8.6) (*p* < 0.05). Moreover, there were significant differences between the mean (SD) of BMI in females with no child 27(5.7), and females with two 29.1(4.8) and three or more children 30.9(5.1) (*p* < 0.05). While there were no significant differences between the BMI of males with different numbers of children (*p* > 0.05) (Table 1). Characteristics of participants were presented in Table 1.

**Table 1:** Characteristics of participants

Characteristic	Female				*P*-value ^d^	Male				*P*-value ^d^
	Number of children					Number of children				
	No (*n* = 65)	One (*n* = 270)	Two (*n* = 1094)	Three and more (n = 1422)		No (*n* = 65)	One (*n* = 270)	Two (*n* = 1094)	Three and more (*n* = 1422)	
**Age, year**^a^	38.5(14.8)^13,14^	39.2(10.7)^23,24^	45.4 (8.1)^34^	53.0(8.5)	< 0.001	44.4(15.4)^13,14^	44.9(10.4)^23,24^	50.6(8.4)^34^	58.1(8.6)	< 0.001
**Smoking status**^c^(Past or current)	7 (11.1)^13,14^	14(5.3)	56(5.2)^34^	43(3.0)	0.002	25(39.1)	130(49.4)	509(48.8)	674(48.8)	0.5
**Education**^c^(Diploma and upper)	37(56.9)^13,14^	126(46.7)^23,24^	424(38.8)^34^	409(28.8)	< 0.001	29(44.6)	131(48.5)^24^	501(45.8)^34^	503(35.4)	< 0.001
**WHtR**^a^	0.56(0.10)^14^	0.57(0.07)^23,24^	0.59(0.07)^34^	0.64(0.08)	< 0.001	0.55(0.08)	0.54(0.06)^23,24^	0.56(0.06)^34^	0.57(0.06)	< 0.001
**BMI** ( kg/m^2^) ^a^	27(5.7)^13,14^	28.0(4.9)^23,24^	29.1(4.8)^34^	30.9(5.1)	< 0.001	27.7(6.6)	26.8(4.3)	27.4(4.2)	27.2(4.1)	0. 1
**Appropriate physical activity**^c^	13(20.3)^12,13^	105(38.9)^24^	372(34.2)^34^	405(28.7)	< 0.001	19(29.2)^13,14^	91(34.5)^23,24^	447(42.0)	571(42.7)	0.02
**SBP**(mmHg) ^a^	113.8(15.1)^14^	110.4(14.4)^24^	111.2(15.1)^34^	119.8(18.8)	< 0.001	123.0(20.0)	117.4(16.0)^24^	119.2(16.1)^34^	125.2(20.0)	< 0.001
**DBP**(mmHg) ^a^	75.4(8.9)	74.9(9.8)^24^	75.7(9.2)^34^	77.9(9.9)	< 0.001	78.5(12.4)	79.1(10.1)	80.0(9.8)	80.0(11.0)	0.4
**FBS** (mg/dl) ^a^	91.9(29.4)^14,24,34^	92.9(17.7)	96.3(26.6)	107.7(39.7)	< 0.001	103.2(30.5)	103.2(35.2)^24^	104.7(34.1)^34^	110.3(40.4)	0.001
**TG** (mg/dl) ^b^	108(84–139)^13,14^	112(78–163)^23,24^	123(87.5–170)^34^	140(101.5–188)	0.001	160(98–205)	156(107–209)	146(105–205)	142(103–197)	0.4
**TC** (mg/dl) ^a^	195.2(48.2)	189.8(40.7)^24^	196.5(40.4)	200.1(42.3)	0.001	192.4(44.7)	193.6(41.5)	192.6(40.4)	190.9(41.0)	0.6
**LDL-C** (mg/dl) ^a^	121.9(36.5)	113.8(34.1)^24^	118.9(34.1)	120.5(37.0)	0.04	118.8(40.9)	118.2(33.9)	117.5(34.1)	116.9(36.1)	0.9
**HDL-C** (mg/dl) ^a^	48.9(12.7)	49.6(11.8)	49.8(11.9)^34^	48.4(11.0)	0.03	41.0(11.3)	40.8(10.8)	41.8(9.8)	41.2(9.9)	0.3
**Incidence HTN**^c^	15(23.1)^14^	59(21.8)^24^	266(24.3)^34^	654(46)	< 0.001	20(30.8)^14^	75(27.8)^23,24^	400(36.6)^34^	687(48.3)	< 0.001
**Ever used blood pressure medication**^c^	6(9.2)^14^	22(8.2)^24^	120(11.0)^34^	352(24.9)	< 0.001	11(16.9)	27(10.0)^24^	143(13.1)^34^	298(21.1)	< 0.001

Abbreviations: *WHtR* Waist-to-Height Ratio, *BMI* Body mass index, *FBS* Fasting blood glucose, *HTN* Hypertension, *SBP* Systolic blood pressure, *DBP* Diastolic blood pressure, *TG* Triglyceride, *LDL-C* Low-density lipoprotein cholesterol, *HDL-C* High-density lipoprotein cholesterol, *TC* Total cholesterol

^a^ Mean (SD)

^b^ median(Q25-Q75)

^c^ n (%)

^d^ ANOVA test, Kruskal–Wallis test, or Pearson's Chi-squared test was used as appropriate; and post hoc comparisons were identified by superscripts 1,2,3,4

1: No child; 2: one child; 3: two children; 4: three and more children

**Fig. 2:**
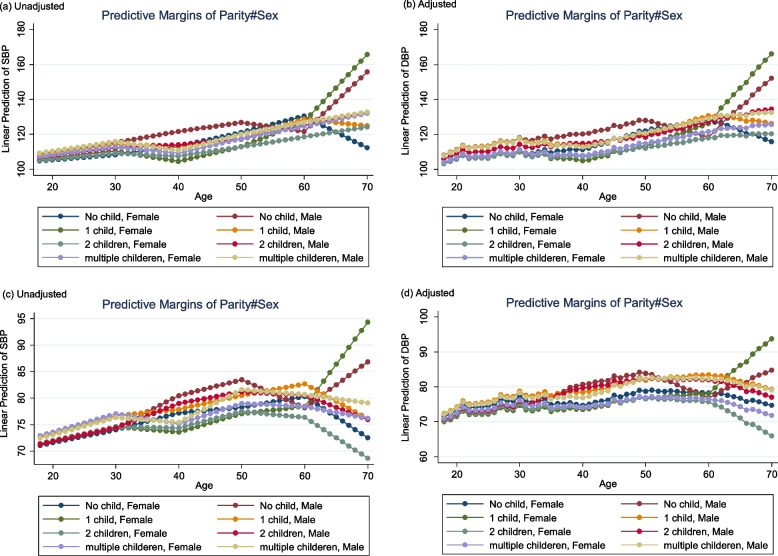
Predictive marginal means plot for SBP (**a** unadjusted, **b** adjusted) and DBP (**c** unadjusted, **d** adjusted) based on different categories of age and parity for males and females obtained from regression spline model

**Fig. 3:**
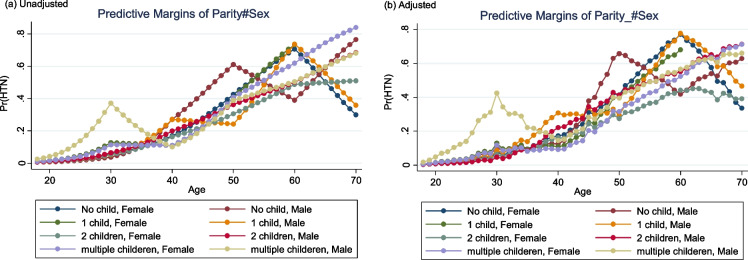
Predictive marginal probability plot for HTN status (**a** unadjusted, **b** adjusted) based on different categories of age and parity for males and females obtained from logistic regression spline model

Figures 2 and 3 show the predictive marginal means (unadjusted and adjusted) of SBP and DBP and the predictive probability plot for HTN status for different age and parity categories (18-70y). Overall, according to the adjusted plot, SBP differed slightly between groups (among males and females aged 18-60y) while the most notable change of adjusted marginal means (AMM) of SBP was observed between the age group of 60-70y among females with one child and males with no child. Moreover, females aged 60-70y with 1 child and females with 2 children experienced the highest and lowest AMM of DBP. Also, we observed differences in terms of a predictive marginal probability for HTN between groups. Young males (18-30y) with multiple children and older males (60-70y) with a child experienced a high probability of HTN.

Table 2 shows unadjusted (superscript a) and adjusted (superscript b) risk ratio (RR) and 95% Confidence Intervals (CIs) for HTN among females and males based on age and parity categories, respectively.

**Table 2:** Risk ratio and 95% Confidence Intervals (CI) for hypertension among female and male in different categorizes of age and parity

**Varible**	**Female**				**Male**			
	**Unadjusted RR(95% CI)**	***p*-value**	**Adjusted RR(95% CI)**	***p*-value**	**Unadjusted RR(95% CI)**	***p*-value**	**Adjusted RR(95% CI)**	***p*-value**
**Aged18-30y**	Ref: no-child		Ref: no-child		Ref: no-child		Ref: no-child	
1 child	1.20(0.96, 1.50)	0.09	1.16(0.94, 1.44)	0.15	1.28(0.67, 2.45)	0.44	1.33(0.67, 2.63)	0.40
2 children	1.17(0.94, 1.46)	0.15	1.14(0.92, 1.41)	0.21	1.29(0.68, 2.42)	0.42	1.31(0.67, 2.54)	0.42
≥ 3 childeren	1.20(0.96, 1.49)	0.09	1.16(0.94, 1.43)	0.14	1.37(0.73, 2.56)	0.31	1.42(0.73, 2.74)	0.29
**Aged:30-40y**	Ref: no-child		Ref: no-child		Ref: no-child		Ref: no-child	
1 child	0.99(0.87, 1.12)	0.88	0.99(0.87, 1.12)	0.92	1.16(1.00, 1.36)	0.04	1.14(0.97, 1.34)	0.09
2 children	1.11(1.01, 1.23)	0.03	1.10(0.99, 1.22)	0.05	1.11(0.99, 1.25)	0.06	1.17(1.02, 1.35)	0.02
≥ 3 childeren	1.01(0.88, 1.14)	0.92	1.00(0.87, 1.14)	0.97	0.88(0.77, 0.99)	0.04	0.87(0.78, 0.99)	0.03
**Aged:40-50y**	Ref: no-child		Ref: no-child		Ref: no-child		Ref: no-child	
1 child	1.14(1.03, 1.25)	0.006	1.14(1.04, 1.26)	0.004	0.99(.92, 1.06)	0.88	0.99(.92, 1.07)	0.92
2 children	1.06(1.02, 1.10)	0.002	1.05(1.01, 1.10)	0.005	1.06(1.01, 1.10)	0.006	1.06(1.02, 1.11)	0.004
≥ 3 childeren	1.13(1.08, 1.19)	< 0.001	1.12(1.07, 1.17)	< 0.001	1.13(1.07, 1.20)	< 0.001	1.11(1.04, 1.17)	< 0.001
**Aged:50-60y**	Ref: no-child		Ref: no-child		Ref: no-child		Ref: no-child	
1 child	1.06 (0.99, 1.14)	0.07	1.06(0.98, 1.14)	0.11	1.12(1.05, 1.19)	< 0.001	1.11(1.05, 1.18)	< 0.001
2 children	1.04(1.01, 1.09)	0.01	1.04(1.01, 1.09)	0.01	1.03(1.01, 1.06)	0.01	1.03(1.00, 1.05)	0.02
≥ 3 childeren	1.04(1.02, 1.06)	< 0.001	1.04(1.02, 1.06)	< 0.001	1.02(1.00, 1.05)	0.03	1.04(1.01, 1.06)	0.001
**Aged60-70y**	Ref: no-child		Ref: no-child		Ref: no-child		Ref: no-child	
1 child	1.05(0.97, 1.14)	0.22	1.06(0.95, 1.20)	0.25	0.92(0.82, 1.035)	0.17	0.95(0.85, 1.07)	0.47
2 children	1.00(0.92, 1.08)	0.95	1.00(0.91, 1.09)	0.98	1.03(.99, 1.06)	0.05	1.04(1.01, 1.08)	0.007
≥ 3 childeren	1.03(1.01, 1.05)	0.001	1.02(1.00, 1.04)	0.01	1.03(1.01, 1.04)	0.001	1.03(1.01, 1.04)	0.002

Adjusted for potential risk factors: BMI, smoking status, physical activity, education, TG and HDL

**Fig. 4:**
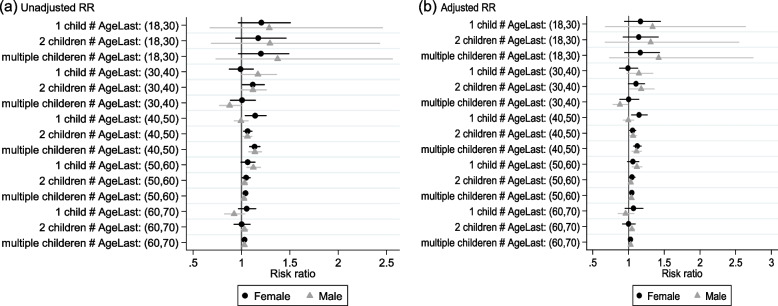
Risk ratio plot of HTN in terms of interaction between Parity number and age in females and males based on (a) unadjusted and (b) adjusted model. Adjusted for potential risk factors: BMI, smoking status, physical activity, education, TG and HDL. The risk ratios (RRs) are plotted on a floating absolute scale. Vertical lines indicate the corresponding 95% confidence intervals (CIs)

Furthermore, in the adjusted model, the risk of HTN in females aged 40-50y with 1 child, 2 and ≥ 3 children compared to no child were 1.14(95% CI, 1.04, 1.26; *P* = 0.004), 1.05(95% CI, 1.01, 1.10; *P* = 0.005), 1.12(95% CI, 1.07, 1.17; *P* < 0.001), respectively. Moreover, in those aged 50-60y with 2 (1.04(95% CI, 1.01, 1.09; *P* = 0.01)) and ≥ 3 children (1.04(95% CI, 1.02, 1.06; *P* < 0.001)), the risk of HTN increased by 4%. Also, in the adjusted model, for females aged 60-70y with ≥ 3 children compared to those without children, the risk of HTN increased by 2% (1.02(95% CI, 1.00, 1.04; *p* = 0.01)). For males aged 30-40y with 2 children compared to no child group, the adjusted risk of HTN increased by 17% (1.17(95% CI, 1.02, 1.35; *P* = 0.02), however for those with ≥ 3 children in the same age group, this risk decreased by 13% (0.87 (95% CI, 0.78, 0.99; *P* = 0.03)). Moreover, males aged 40–50 years with 2 (1.06(95% CI, 1.02, 1.11; *P* = 0.004)) and ≥ 3 (1.11(95% CI, 1.04, 1.17; *p* < 0.001)) children showed an increasing risk of HTN compared to no child in the same group. Also, in males aged 50–60 y, we found that having a child was associated with an increased risk of HTN. Additionally, in males aged 60-70y, we found that compared to the childless males, the risk of HTN was 1.04 (95% CI, 1.01, 1.08; *p* = 0.007) and 1.03(95% CI, 1.01, 1.04; *p* = 0.002) in those males with 2 and ≥ 3 children, respectively (Table 2 and Fig. 4).

In the unadjusted GLM model, the risk of HTN increased by 19% in males than females (RR: 1.19; 95% CI: (1.11, 1.27), *p*-value < 0.001). The adjusted model also showed that the risk of HTN increased by 13% in males than in females (RR: 1.13; 95% CI: (1.05, 1.23), *p*-value = 0.002).

## Discussion

This study attempted to examine whether parity provokes an impact on HTN in couples with different age groups. In this population-based study, we found that regardless of age group, higher parity (≥ 3 parity) was associated with a higher risk of HTN among both females and males. After adjustment for potential risk factors, among the females aged 60-70y with one child, SBP increased compared to childless females. While for males aged 60-70y, having children significantly decreased the mean of SBP compared to the childless males. Meanwhile, childless females or those with one child had higher SBP than the males in either group (60-70y). In terms of prediction DBP, there were no significant differences between the groups. Differences in mean SBP and DBP within the age categories were generally negligible.

Nowadays, the BP trajectory has an upward trend in low- and middle-income countries [14]. Regardless of this trajectory, HTN is a well-known common risk factor for CVDs, CKD, and associated events [25, 26]. Additionally, HTN and elevated SBP are the leading global burden of NCD risk factors and the causes of mortality worldwide [27, 28]. Multiple genetic, epigenetic, environmental, and social factors are determinants of HTN [29]. There is a growing number of evidence that highlights the role of reproductive history on health later in life [30, 31]. From the biological point of view, reproduction in a female’s life is costly in terms of physiological adaptation [32]. The exact underlying mechanism of association of HTN and the number of parity remains poorly understood. Part of this association could be explained by biological modifiers related to the physiological alterations related to normal pregnancy; this association could be exacerbated by pregnancy abnormalities [8, 33]. Becoming a parent could change the female’s and male’s life in both positive and negative ways [34]. Moreover, among males, the non-pregnancy-related pathway contributes to the underlying pathway of association between parity and mortality [35]. Hence, the number of parity could influence SBP, DPB, and HTN in both males and females via different pathways.

We found that females aged 60-70y without a child and females with one child had higher SBP than the males in either group. The association between parity and HTN has been reported in several previous studies [16–18] with inconclusive results. However, limited studies have comprehensively examined the association between parity and BP in both males and females. In line with our work, a previous study on 133 African women found that parity > 5 could act as a risk factor for HTN [16]. Additionally, in the Iranian population, the result of a study showed that women with ≥ 3 parity are at increased risk for HTN [18]. Another study demonstrated that HTN among Norwegian males and females aged 40-80y without a child or with a child was more common than among females and males with 2 children [36]. However, in contrast to our study, an Italian study reported that parity was not associated with HTN during postmenopausal [17]. In a previous study, the association between parity and HTN and metabolic syndrome in postmenopausal women was not confirmed. It is proposed this is related to the physiological changes related to menopause and ageing to some extent [13], while Moazzeni et al. (2020) found a J-shaped association between parity and CVD [37]. The impact of parenting on the overall health of males and females may be influenced by several factors including gender, women’s role in the decision-making process, and family unit. Moreover, ordinary risk factors including smoking, diet, and physical activity play a role [38–40].

Our result showed that for males aged 60-70y, having children significantly decreased the mean of SBP compared to the males without a child. However, among females aged 60–70 years with one child, SBP increased compared to the females without a child. It is well known that subfertility and nulliparity per se are known as risk factors for CVD in women [41]. The great impact of parenting, especially at an older age, may provide a sense of security and support for parents which could decrease their feeling of loneliness [42]. Evidence shows the association of nulliparity and low parity with poor health behaviors, lack of social support, and subsequent adverse health outcomes [43]. Moreover, findings from the British Women’s Heart and Health Study and the British Regional Heart Study revealed that the prevalence of CHD in women and men with no child or only one child increased [44]. It should be noted that some childless couples might suffer from reproductive-related disorders (such as infertility, experiencing complications of pregnancy, polycystic ovary syndrome, etc.). Some of these medical conditions may also adversely their general health status [44, 45]. Evidence showed that, there is regional fertility difference in different locations among fertile couples [46, 47]. What is more, the regional differences in reproductive patterns can impact of the association between parenthood and cardio-metabolic risk factors [48]. It should be noted that sex differences could also contribute to explaining the underlying mechanism in differences in HTN in both males and females. In this study, females were younger than males and their smoking status, lipid profile, and FBS were more favorable than males.

Further, the result of a study on the rural women of Bangladesh demonstrated that in females with 1 parity, DBP was lowest; but, in females with > 2 parity as well as in females without parity, DBP was elevated. This association increased in females without parity after 45y [49]. However, in our study, there was no significant association between parity and DBP. The results also showed that, among males, as parity and age increased, the adjusted marginal means of SBP and DBP increased. However, among childless males and females aged 60-70y, the mean of SBP and DBP was higher than that among males and females with one child. A Swedish study evaluated the metabolic profile of childless males and observed an elevated risk of CVD [50]. Married males without children, which might reflect infertility conditions, demonstrated a higher risk of cardio-metabolic diseases [51]. The diversities in these studies might be due to the differences in the classification of variables and adjustment for relevant characteristics and other methodology-related factors. In light of the high smoking rate among males, it is possible that the effect of parity was obscured by the influence of smoking on BP. Beyond the physiological changes of pregnancy in females and the interplay of genetic and environmental factors in both sexes, factors related to childrearing could play a role in the later health of parents [43].

Parenthood across different stages of life can strain psychological well-being and may influence health [34]. A recent study demonstrated that the effect of ageing posed a greater effect on SBP and DBP of males than the females in age groups 40–49 and 30–39 years [52]. Healthy behavior in parenthood during different life stages may face a paradox. Additionally, lifestyle risk factors related to the child-rearing process may contribute to the formation of an obesogenic environment and subsequent CVD risk in both males and females [44]. Obligations related to promoting the overall well-being of children, providing for their financial demands, and planning family diets based on the interests of children’s needs could result in promoting or deterring effects on the parents’ health [53]. Every lifetime stress exposure (such as stress related to the low socioeconomic status, occupational stress, and stressful aspects of the social environment) act as risk factor for HTN [54]. Alternatively, socioeconomic and lifestyle-related factors associated with HTN risk might differ between both sexes, and different age groups across various categories of the number of parity.

Nonetheless, this study has some limitations and strengths that need to be considered in conjunction with the results. The main strength of the present study is its methodology which uses a population-based study data set with a large sample size of couples and reliable assessment of variables. Furthermore, the cardio metabolic variables were measured directly. In this study, we included different age range of couples with various number of children. Our results were less likely to be influenced by selective recall bias since the distribution of the number of children for both sexes was similar. Another limitation of this study is that we did not have information on the genetic factors and some important lifestyle factors affecting the risk of HTN. This study was also limited by not considering some influential factors such as nutrition, economic situation, family norms, and psychosocial health. This study was restricted to only married individuals, and widowed or divorced persons were not included.

In this study, we have considered the effect of higher BMI in the adjusted models. Besides that, the effect of obesity on HTN in both males and females was significant. One of the well-recognized risk factors for HTN is obesity. In fact obesity via enhancing the activity of renin–angiotensin–aldosterone system and the sympathetic nervous system [55]. It seems that biological changes related to pregnancy and lifestyle factors could affect the risk of developing metabolic syndrome components in women [56].

Further comprehensive well designed longitudinal studies assessing various influential factors are recommended to investigate the possible mechanisms linking parity and HTN. More frequent HTN screening would be advisable among childless couples. Identifying and preventing HTN cases would be highly impactful. History taking of number of children and representing the consulting program for couples is a potentially affordable and cost-effective approach for the prevention of HTN. This approach especially in low and middle-income countries where HTN remains largely undiagnosed and uncontrolled is the most cost-effective.

## Conclusion

We observed that, in both males and females, having children and being childless were associated with HTN. Our study presented novel findings for the association between parity and HTN among couples with various numbers of parity within different age groups. As HTN and elevated BP are the leading causes of mortality in males and females, it is increasingly important to develop a strategy for health promotion and disease prevention. Recognizing the potential association of parity with HTN could help identify high-risk couples.

## Data Availability

The datasets used and/or analyzed during the current study available from the corresponding author on reasonable request.

## Abbreviations

HTN: Hypertension
CVD: Cardiovascular diseases
CKD: Chronic kidney disease
FBS: Fasting blood glucose
TG: Triglyceride
LDL-C: Low-density lipoprotein cholesterol
HDL-C: High-density lipoprotein cholesterol
TC: Total cholesterol
BP: Blood pressure
SBP: Systolic blood pressure
DBP: Diastolic blood pressure
